# T-Cell Leukemia/Lymphoma 1 (TCL1): An Oncogene Regulating Multiple Signaling Pathways

**DOI:** 10.3389/fonc.2018.00317

**Published:** 2018-08-13

**Authors:** Francesco Paduano, Eugenio Gaudio, Afua A. Mensah, Sandra Pinton, Francesco Bertoni, Francesco Trapasso

**Affiliations:** ^1^Dipartimento di Medicina Sperimentale e Clinica, University Magna Græcia, Catanzaro, Italy; ^2^Biomedical Section, Tecnologica Research Institute, Crotone, Italy; ^3^Institute of Oncology Research, Università della Svizzera italiana, Bellinzona, Switzerland

**Keywords:** T-cell leukemia/lymphoma 1A (TCL1A), TCL1 interacting partners, CLL and lymphomas, TCL1-positive hematological malignancies, targeted therapy

## Abstract

Almost 30 years ago, Carlo Croce's group discovered the T-Cell Leukemia/Lymphoma 1A oncogene (*TCL1A* or *TCL1*). TCL1 protein is normally expressed in fetal tissues and early developmental stage lymphocytes. Its expression is deregulated in chronic lymphocytic leukemia (B-CLL) and most lymphomas. TCL1 plays a central role in lymphomagenesis as a co-activator of AKT kinases and other recently elucidated interacting protein partners. These include ATM, HSP70 and TP63, which were all confirmed as binding partners of TCL1 from co-immunoprecipitation experiments utilizing endogenously expressed proteins. The nature of these interactions highlighted the role of TCL1 in enhancing multiple signaling pathways, including PI3K and NF-κB. Based on its role in the aforementioned pathways and, despite the lack of a well-defined enzymatic activity, TCL1 is considered a potential therapeutic target for TCL1-positive hematological malignancies. This perspective will provide an overview of TCL1A and its interacting partners.

## T-cell leukemia/lymphoma 1A (TCL1A)

*TCL1A* is an oncogene that was discovered by Carlo Croce's group during the 1980's (1–4). Its product, TCL1, is a 13 kDa protein whose function requires it to form homodimers (4). TCL1 acts as co-activator of AKT kinases and when physiologically expressed it mediates normal growth and survival signals, while when dysregulated, it causes lymphomagenesis and cancer progression (4–7). TCL1 is the key isoform of the TCL1 family proteins that are involved in the normal development of early B- and T-cells (4).

The expression of TCL1 has been described in germinal center (GC) centroblast, centrocyte and post-GC memory B cells, in tumors arising from the germinal center such as follicular lymphoma (FL), Burkitt lymphoma (BL), diffuse large B cell lymphoma (DLBCL) and from memory cells such as chronic lymphocytic leukemia (CLL) (4, 8). Prolonged and increased expression of TCL1 in the late phases of thymocyte development causes T cell prolymphocytic leukemia (T-PLL) (3, 4). TCL1 dysregulation in T cells is due to a chromosomal translocation that brings *TCL1* (on chromosome 14q31.2) under *TCR* (T Cell Receptor) enhancer elements. The precise mechanisms underlying the over-expression of TCL1 in B cell tumors are unclear, since neither translocations nor Epstein-Barr Virus (EBV) infection are involved (9, 10). Transcriptional events and altered epigenetic signals might underlie the aberrant expression of TCL1 in B cell malignancies (11, 12).

The oncogenic profile of TCL1 has been studied using *in vitro* (e.g., hematological cancer cell lines and primary B- and T-cells) and *in vivo* models. Two murine *TCL1* transgenic models have been generated (13). Virgilio and colleagues generated a Lck-*TCL1* transgenic mouse model that develops T cell leukemias (14), while Bichi and colleagues generated the E(mu)-*TCL1* transgenic mouse that develops leukemia resembling the aggressive form of human CLL (15). These *TCL1* transgenic mice have been widely used by the scientific community, especially to model human CLL and evaluate novel anti-lymphoma compounds.

## TCL1 interacting partners

Laine and colleagues were the first to demonstrate a physical interaction between TCL1 and AKT and proposed a mechanism by which TCL1 enhances AKT phosphorylation with consequent phosphorylation of downstream targets and increases in cell proliferation and survival (5). In a yeast two-hybrid screen where AKT2 was used to screen an EBV-transformed B cell cDNA library, AKT2 was also identified as a putative TCL1-interacting partner (5, 6, 16).

Successively, several publications have described additional proteins that interact with TCL1. These include in particular, work performed by French and colleagues that elucidated the interaction between TCL1 and PNPase, a polynucleotide phosphorylase (3′-5′ exoribonuclease) (17). They enriched potential TCL1 partners via immunoprecipitation, successive coomassie staining and mass spectrometry resolving analysis and corroborated these results with molecular docking data.

**Figure 1:**
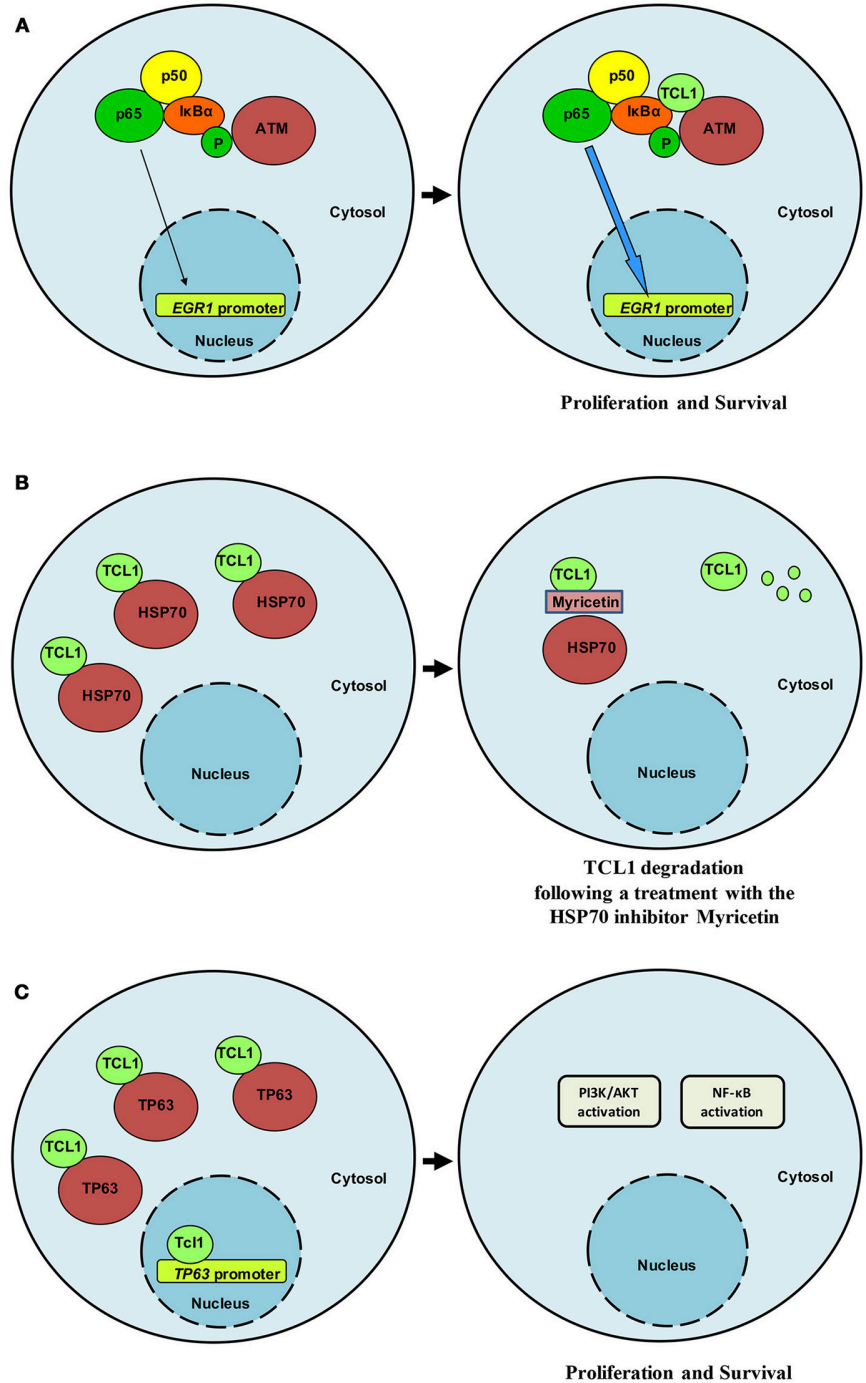
TCL1 interacts relevant partners. **(A)** TCL1 interacts with ATM and IκB and induces enhanced activation of the NF-κB signaling pathway in CLL and lymphoma. **(B)** HSP70 regulates TCL1 protein folding and stabilizes its expression. Following treatment with myricetin, HSP70 activity is compromised and TCL1 is ubiquitinated and degraded. **(C)** TCL1 interacts with the transcription factor p63 and with its promoter region by recognizing an AP1 consensus sequence. TCL1 and p63 cooperate to activate both the PI3K and the NF-κB signaling pathways.

Interest in TCL1 heightened following discoveries that uncovered its interaction with notable proteins such as IκB (18), ATM (19), HSP70 (20), DNMT3A (21), ROR1 (22), and TP63 (23). Pekarsky and colleagues demonstrated that TCL1 was able to interact with several exogenously overexpressed proteins in immunoprecipitation experiments, including EP300/CREBBP, c-JUN, JUN-B and c-FOS (24). Despite these interactions not being confirmed in the context of endogenously expressed proteins, luciferase assays indicated that TCL1 overexpression causes CLL by directly enhancing NF-κB activity and inhibiting AP-1, strengthening the likelihood of effective protein-protein interactions between endogenous proteins and supporting a cross-talk between TCL1 and elements of survival and proliferative pathways. Furthermore, TCL1 expression has been shown to correlate with NF-κB activation in a series of 600 CLL samples (24). Running a yeast two-hybrid screen, Ropars and colleagues found that TCL1 was able to bind directly to the ankyrin domain of IκB, the inhibitor of the NF-κB transcription factor. The interaction was demonstrated by using recombinant proteins overexpressed in HEK293 cells. This discovery represented an important bridging point from which we were able to define the conjunct role of TCL1 and ATM in the NF-κB pathway. Using a proteomic approach to study the oncogenic functions of TCL1 in Burkitt lymphoma and uncover the mechanism of action behind each protein-protein interaction, we identified ATM (19), HSP70 (20), and TP63 (23) as TCL1-interacting proteins. We thus confirmed the direct involvement of TCL1 in the regulation of the NF-κB pathway, as previously suggested by others (18, 24). TCL1 together with ATM and IκB, is responsible for enhancing NF-κB signaling (19). The association of ATM with TCL1 causes enhanced IκB alpha phosphorylation and ubiquitination and subsequent activation of the NF-κB pathway (19). The direct phosphorylation of IκB carried out by ATM is strongly pronounced in the presence of TCL1 (Figure 1A). As a consequence of a prolonged and stronger phosphorylation of IκB, activated NF-κB, represented by p65 (REL-A), translocates to the nucleus where it directly binds the *EGR1* promoter region (19). The final result of this mechanism of action includes increased proliferation and survival. Biological experiments proved the role of TCL1 in NF-κB activation both in human and murine cellular samples. In human B-CLL primary cells, EGR1 protein expression and ATM activity are affected by knocking down TCL1 expression with siRNA and ATM activity with the ATM inhibitor Kudos 55933. Finally, B-cells isolated from transgenic *TCL1* murine spleens show an ATM expression correlated with age and disease development.

In the same year that we reported the TCL1-ATM interaction, two additional studies describing novel TCL1 interactors were published. The first study used Tg *TCL1* mice to validate the interaction between TCL1 and XBP1, a component of the IRE1/XBP1 pathway responsible for the reticulum stress response mechanism. TCL1 overexpression induced an upregulation of XBP1 and also indirectly upregulated other transcriptional factors, such as IRF4, BLIMP1 and AID (25). The chemical inhibition of XBP1 caused the death of CLL cells in both *in vitro* and *in vivo* models. These observations indicate that a combined therapeutic approach hitting both TCL1 partners and TCL1 itself could represent an active therapeutic approach against TCL1-associated malignancies. The second study reported DNMT3A and DNMT3B (DNA methyltransferase 3A and DNA methyltransferase 3B) as putative TCL1 partners. The authors transfected HEK293 cell with GST-*TCL1* and performed a pull-down of all proteins interacting with TCL1, successively resolved by LC/MS-MS. The interaction was confirmed by immunoprecipitation of exogenously overexpressed TCL1 and DNMT3 proteins. The biological rationale for the interaction between TCL1 and DNMT3 proteins was provided by analysis of primary CLL samples and by a transgenic mouse model. This revealed that most CLL samples with high expression of TCL1 showed reduced DNA methylation in comparison to CLL samples with low TCL1 expression. The enzymatic activity of DNMT3 was evaluated by measuring the incorporation of tritium-labeled AdoMet (S-adenosyl-L-[methyl-3H] methionine by 1 mg of DNA substrate (21, 26). The transgenic model presented a strong TCL1 expression that correlated with the inhibition of DNMT3A function. Additionally, in isolated B cells, the correlation between TCL1 expression and DNA methylation overlapped the observations made in primary human CLL samples (21).

TCL1 is also involved in regulating of the promoter of the gene encoding protein tyrosine phosphatase RO truncated (PTPROt), a tumor suppressor that is downregulated in both human CLL and transgenic *TCL1* mice. Luciferase experiments demonstrated a direct role of TCL1 in the down-regulation of *PTPROt* promoter activity along with AP1 elements c-FOS and c-JUN (27), further validating the connection between TCL1 and transcription factors components of the AP1 complex. TCL1 modulates the promoter of the tumor suppressor *PTPROt* and, consequently, the activity of BCR pathway, negatively regulated by PTPROt.

The interaction between TCL1 and the receptor tyrosine kinase-like orphan receptor 1 (ROR1) strongly activates the PI3K/AKT/mTOR pathway and amplifies leukemogenesis in Tg *TCL1* × Tg *ROR1* crossed mice (22, 28). Further work by Daneshmanesh et al. in primary CLL samples treated with antibodies directed toward ROR1 enforced the role of TCL1 as a co-activator of the PI3K/AKT/mTOR pathway via direct contact with AKTs and indirectly through ROR1 (29).

Successive findings proposed a central role for TCL1 in the transcriptional activity of TP63 (23) and uncovered a means of indirectly affecting TCL1 expression and activity. TCL1 physically interacts with Heat Shock Protein 70 (HSP70) and the latter regulates TCL1 protein folding with consequent stabilization of TCL1 (20) (Figure 1B). Inhibition of the ATPase activity of HSP70 by using the inhibitor myricetin results in the ubiquitination and proteasome-dependent degradation of TCL1 (20). Additionally, *in vivo*, the anti-oxidant myricetin significantly reduces lymphoma xenografts and TCL1 expression Overall, these findings suggest that TCL1 is a novel client protein of HSP70 and that the chemical inhibition of HSP70 could represent an indirect way to inhibit TCL1 through its degradation. We have shown that myricetin increases *TP53* promoter activation by blocking HSP70 activity (20) in accordance with Zylicz and colleagues who reported how HSP70 impairs p53 functions (30).

**Figure 2:**
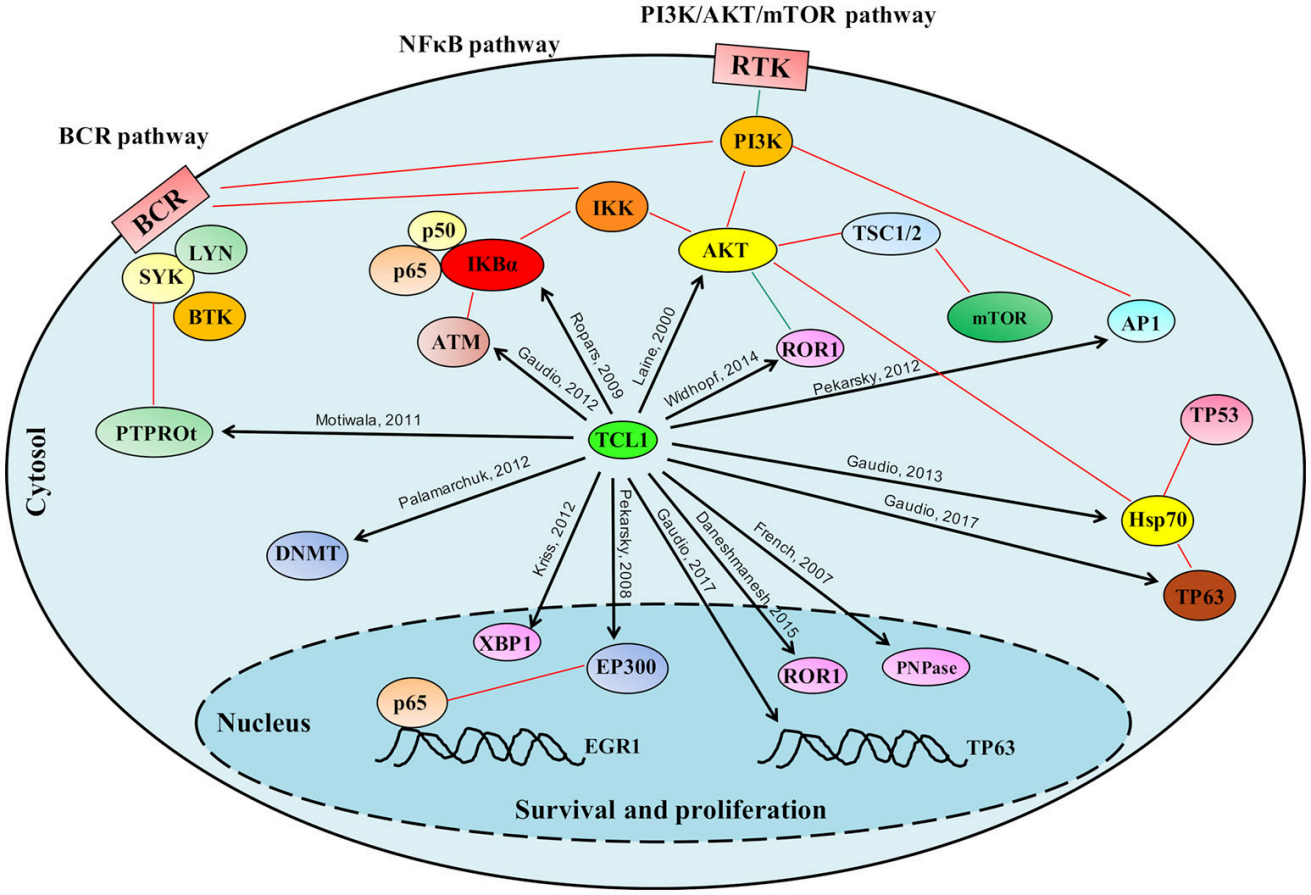
Schematic representation of the role played by TCL1 in multiple signaling pathways. TCL1 regulates many proteins responsible for cellular proliferation, survival and epigenetic regulation through multiple signaling pathways. TCL1 promotes the activation of the PI3K/AKT/mTOR pathway directly through its interactions with AKT, AP1 elements (c-JUN, c-FOS), and ROR1, and indirectly by inhibiting the tumor suppressor PTPROt and sustaining the BCR pathway. TCL1 enhances the activation of the NF-κB pathway by direct actions on IκB alpha and ATM. Other signaling pathways mediated by physical interactions of TCL1 with p63 and HSP70 or with EP300 supports the PI3K/AKT/mTOR and the NF-κB pathways, respectively. Black arrows indicate the protein-protein interactions described in the text; Red lines represent published interactions among proteins and pathways.

For the p53 family member, p63 successive experiments confirmed binding of TCL1 to this protein as well as to its promoter region at an *AP1* consensus sequence (Figure 1C). Concomitant knockdown of TCL1 and TP63 affected AKT phosphorylation status in the Burkitt lymphoma cell line Raji, an observation that fits well with the model we proposed in which TCL1 plays a central and crucial role in multiple signaling pathways (Figure 2).

## TCL1 as a therapeutic target

TCL1 is emerging as a new target for anticancer therapy because of its involvement in important signaling pathways that are up-regulated in hematologic malignancies, as described in the previous section. The available literature describing the biology, functions and structure of TCL1 are convincing academic researchers and biotechnology companies to attempt the synthesis of chemical inhibitors. However, there are two considerations to be made: (1) TCL1 does not exert clear and known enzymatic activity; (2) only the homodimer is active and works as a co-activator of AKT and presumably of all other listed partners. While the first point does not provide any targetable region in the protein, the second does. The region responsible for homodimerization and in particular the amino acid most directly involved in dimerization are well-known (16). Targeting this region with a specific ligand that prevents homodimerization could effectively inhibit TCL1 co-activator function.

In general, two main approaches leading to the identification of TCL1 inhibitors could be undertaken. The first one is based on phage display technology (31, 32) to identify TCL1 antagonist peptides. This strategy is supported by a number of observations. Peptides isolated from the TCL1 protein (very high homology to TCL1 amino acid sequence) show potential activity (33). A TCL1 peptide called AKT-in, spanning a region of TCL1, can inhibit AKT, confirming the crucial role of TCL1 as an activator of AKT and proposing the peptide itself as a therapeutic agent. We believe that the proof of concept represented by a peptide with antagonist effect toward TCL1 should be actually translated in a mimetic drug-like chemical. Peptide-mimetics once generated would have a greater chance of targeting TCL1 with increased efficacy, accuracy and stability compared to peptides. TCL1 peptides for immunotherapy have recently been patented (patent WO 2013075105 A3); they bind to MHC I (HLA-A2) on tumor cells or other antigen-presenting cells and are recognized by T-cell receptors on T cells (34). Related to this, Weng et al published an interesting study describing an epitope of TCL1 that spans amino acid residues 71–78, and is recognized by cytotoxic T lymphocytes. This epitope therefore represents a lymphoma-associated antigen that can be used for the generation of new immunotherapeutic agents against leukemia and lymphomas (34).

The second approach to identify TCL1 inhibitors is based on drug discovery methods that identify lead compounds to be successively improved. The crystal structure of human TCL1 (35) can be used as a starting point for the computational search of ligands/inhibitors for which the chemical feasibility is known. The next step involves the *in silico* screening of large libraries of chemical compounds provided by available public depositories such as Zinc (36), PubChem (37), and MMsINC (38). The molecules identified as able to bind to TCL1 *in silico* could be further validated by assessing their ability to impede the formation of TCL1-TCL1 homodimers *in vitro*. Cells expressing TCL1 would be treated with a selection of the compounds identified from the *in silico* analysis. Protein lysates from these cells would be analyzed by Western blotting in non-denaturing conditions to allow discrimination between homodimers and monomers of TCL1.

As TCL1 inhibitors are yet to be discovered, this approach currently represents a feasible and important avenue of investigation to be pursued for the generation of novel small molecules for the treatment of TCL1-driven hematological malignancies.

## Author contributions

FP, EG, AAM, SP, FB, and FT conceived and wrote the manuscript.

### Conflict of interest statement

The authors declare that the research was conducted in the absence of any commercial or financial relationships that could be construed as a potential conflict of interest.
